# Deep Tumor‐Penetrated Nanocages Improve Accessibility to Cancer Stem Cells for Photothermal‐Chemotherapy of Breast Cancer Metastasis

**DOI:** 10.1002/advs.201801012

**Published:** 2018-10-29

**Authors:** Tao Tan, Hong Wang, Haiqiang Cao, Lijuan Zeng, Yuqi Wang, Zhiwan Wang, Jing Wang, Jie Li, Siling Wang, Zhiwen Zhang, Yaping Li

**Affiliations:** ^1^ State Key Laboratory of Drug Research & Center of Pharmaceutics Shanghai Institute of Materia Medica Chinese Academy of Sciences Shanghai 201203 China; ^2^ School of Pharmacy Shenyang Pharmaceutical University Shenyang 110016 Liaoning China

**Keywords:** cancer metastasis, cancer stem cells, deep tumor penetration, drug delivery, nanocages

## Abstract

Cancer stem cells (CSCs) are proposed to account for the initiation of cancer metastasis, but their accessibility remains a great challenge. This study reports deep tumor‐penetrated biomimetic nanocages to augment the accessibility to CSCs fractions in tumor for anti‐metastasis therapy. The nanocages can load photothermal agent of 1,1‐dioctadecyl‐3,3,3,3‐tetramethylindotricarbocyanine iodide (DBN) and chemotherapeutic epirubicin (EBN) to eradicate CSCs for photothermal‐chemotherapy of breast cancer metastasis. In metastatic 4T1‐indcued tumor model, both DBN and EBN can efficiently accumulate in tumor sites and feasibly permeate throughout the tumor mass. These biomimetic nanosystems can be preferentially internalized by cancer cells and effectively accessed to CSCs fractions in tumor. The DBN+laser/EBN treatment produces considerable depression of primary tumor growth, drastically eradicates around 80% of CSCs fractions in primary tumor, and results in 95.2% inhibition of lung metastasis. Thus, the biomimetic nanocages can be a promising delivery nanovehicle with preferential CSCs‐accessibility for effective anti‐metastasis therapy.

## 1 Introduction

Cancer stem cells (CSCs), also known as tumor‐initiating cells, have been proposed to account for the tumor origin, long‐term growth, metastasis, and relapse after therapy.^1^, ^2^ Especially for cancer metastasis, compelling evidences suggest that CSCs are responsible for the initiation of metastasis and may be the only cells capable of establishing metastatic tumors in distant organs.^3^, ^4^ Rationally, eradicating CSCs in dormant tumor can be a conceivable strategy to prevent their metastasis to distant organs.

To date, only a few therapeutic agents are used alone or their combinations to eradicate the CSCs,^2^, ^5^, ^6^, ^7^ but their applications largely suffer from the poor delivery efficiency to CSCs. It is worth noting that breast CSCs are the only minor subset of cancer cells in the heterogeneous solid tumor.^2^, ^8^ In addition to the perivascular niche, the aldehyde dehydrogenase (ALDH) expressing breast CSCs can reside in hypoxic niche distal from tumor vasculature and locate more centrally in the tumor internal zones.^9^ Moreover, the breast CSCs are supported by tumor‐associated macrophages (TAM) and cancer‐associated fibroblasts (CAF) as well as multiple extracellular matrix (ECM) components, forming an abominable barrier hampering drug accessibility to CSCs.^10^, ^11^ Unarguably, it is highly desired to find novel substantial strategies to improve the accessibility of therapeutic agents to CSCs for anti‐metastasis therapy.

Nanosystems can effectively deliver various therapeutic agents to tumor sites, providing an essential prerequisite to augment their accessibility to CSCs.^6^, ^12^, ^13^ However, due to the rarity of CSCs, high heterogeneity of tumor mass, and dense network of ECM, many current nanosystems are usually trapped in the matrix just outside tumor vessels and largely inadequate to permeate into the deep interior region of tumor mass.^10^, ^14^, ^15^ The electron microscopy results suggest that the openings in ECM are generally less than 40 nm, which would inevitably hinder the diffusion of large nanoparticles in tumor.^16^ The smaller‐sized nanoparticles display superior penetrating capability in tumor mass, but they may be extensively hijacked by TAM and CAF upon their intratumoral transport,^17^, ^18^ thereby significantly compromising their accessibility to CSCs in deep tumor regions. To circumvent this dilemma, nanosystems should be endowed with outstanding capabilities of tumor accumulations, deep tumor penetration, specific recognition, and effective accessibility to CSCs.^2^, ^9^, ^12^, ^15^, ^19^

Intriguingly, bioinspired strategies have attracted increasing attentions for deep tumor‐penetrated drug delivery and unique accessibility to cancer cells owing to their tumor‐homing ability and biomimetic biological properties.^20^, ^21^ Ferritin is a ubiquitous endogenous iron storage protein nanoparticles and presents as a cage‐like nanostructure with the outer diameter of 12 nm and inner cavity of 8 nm, which can load a large variety of therapeutic agents for tumor imaging and therapy.^22^, ^23^ Moreover, ferritin can specifically bind to the highly expressed transferritin receptor 1 (TfR1) and scavenger receptor class A membrane 5 (Scara5), potentiating it an encouraging nanoplatform for efficient targeting and deep penetration in tumor.^23^, ^24^, ^25^, ^26^ Recently, Rich and co‐workers demonstrate that ferritin can be preferentially required by CSCs in brain tumor to facilitate the propagation and tumorigenicity in vivo.^26^ Based on this rationale, the ferritin nanocages could be an ideal biomimetic nanovehicle to deliver various therapeutic agents to CSCs in tumor masses for effective anti‐metastasis therapy.

**Scheme 1:**
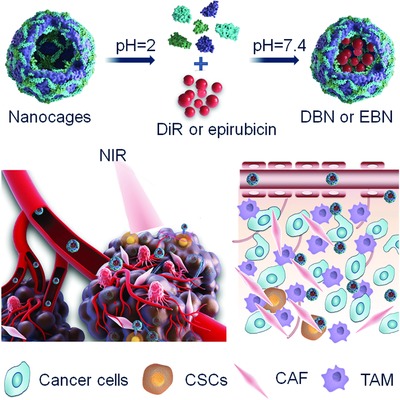
Schematic illustration of deep tumor penetrated biomimetic nanocages with preferential CSCs‐accessibility for effective anti‐metastasis therapy. The biomimetic nanocages can effectively deliver photothermal agents of DiR and cytotoxic epirubicin to CSCs in tumor mass for photothermal‐chemotherapy of breast cancer metastasis.

For this perspectives, we herein explored the biomimetic nanocages of apoferritin as a nanovehicle loading a near‐infrared (NIR) dye of 1,1‐dioctadecyl‐3,3,3,3‐tetramethylindotricarbocyanine iodide (DiR) and cytotoxic epirubicin to improve their accessibility to CSCs in tumor for combinational therapy of breast cancer metastasis (**Scheme** **1**). The specific recognition of DiR‐loaded biomimetic nanocages (DBN) by CSCs were investigated in a CSCs‐enriched 3D tumorsphere model. Importantly, the in vivo deep tumor penetration of DBN and epirubicin‐loaded biomimetic nanocages (EBN) as well as their preferential accessibility to CSCs in tumor were underscored in a metastatic 4T1‐induced breast cancer model. Moreover, the therapeutic effects on eradiating CSCs and suppressing lung metastasis were examined to validate the feasibility of this CSCs‐assessing strategy on treating breast cancer metastasis.

## 2 Results and Discussion

### 2.1 Characterization of DBN and EBN

**Figure 1:**
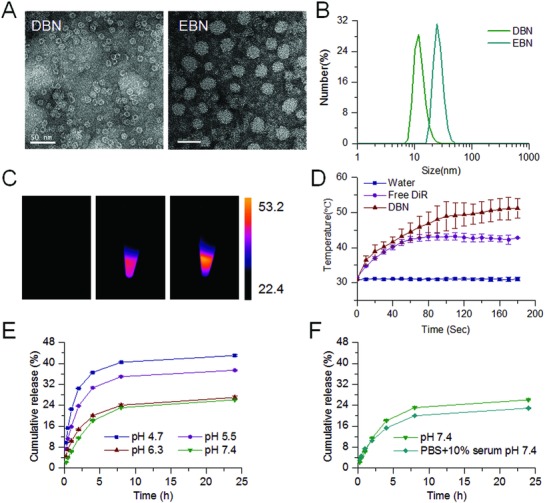
Characterization of DBN and EBN. A) Typical TEM images of DBN and EBN, scale bar = 50 nm. B) The particle size distribution of DBN and EBN measured by DLS analysis. C) The thermal images of water, free DiR, and DBN upon their exposure to an 808 nm laser. D) The in vitro temperature variations of water, free DiR, and DBN upon 808 nm laser irradiation. E) The in vitro release profiles of EBN in PBS at different pH values. F) The in vitro release profiles of EBN in PBS (pH 7.4) and PBS (pH 7.4)+10% FBS.

Initially, the biomimetic nanocages of DBN and EBN were respectively prepared by a disassembly–assembly process using the pH variations of the solution from 2 to 7.4. The nanocages would disassemble into subunits in acidic environments (pH 2), and intactly restore into nanocages upon the pH value back to 7.4, allowing the flexible encapsulation of various therapeutic agents into the nanocages. Additionally, the ferritin protein nanocages are heat‐stable, even when the temperature is up to 85 °C,^27^ which could be used to load DiR for photothermal therapy. The morphologies of DBN and EBN were using biology transmission electronic microscope (TEM), while the particle size was determined by dynamic light scattering (DLS) analysis. The TEM images showed that DBN presented as cage‐like nanoparticles with the mean diameter of 12.22 ± 2.77 nm, while EBN were homogeneous spherical particles with the mean diameter of 26.03 ± 5.03 nm (**Figure** **1**A,B). Accumulating data have evidenced the superior ability of small‐sized nanoparticles for deep tumor‐penetrated drug delivery.^18^, ^28^, ^29^ The small‐sized biomimetic nanocages of DBN and EBN would provide a feasible necessity to improving their accessibility to CSCs in tumor interior regions.

The quantified results showed the loading capacity in nanocages was 1.04% for DiR in DBN and 13.34% for epirubicin in EBN. The difference in morphology and particle size between DBN and EBN could be presumably ascribed to their distinct loading capacity in the nanocages. Then, the heat‐generating ability of DBN upon laser irradiation was monitored using a thermal camera (Figure 1C,D). Upon their exposure to an 808 nm laser at 2.0 W cm^−2^ for 3 min, the temperature could be significantly increased to 51.2 ± 2.8 °C in DBN and moderately enhanced to 42.8 ± 1.1 °C in free DiR. These results confirmed the dramatic enhancement of DiR‐mediated photothermal effects after their incorporation into DBN. Afterward, the in vitro release profiles of epirubicin from EBN were investigated in phosphate buffered solutions (PBS) at different pH values or in PBS (pH 7.4) containing 10% fetal bovine serum (FBS) (Figure 1E,F). In PBS at different pH values, epirubicin was released from EBN in a pH‐responsive manner (Figure 1E). The drug release of EBN in PBS at pH 5.5 and 4.7 could rapidly increase to about 40% within 24 h, which was much higher than that in PBS at pH 6.3 and 7.4 at certain time intervals. Additionally, there was no significant difference between the release profiles of EBN in PBS (pH 7.4) and PBS (pH 7.4) + 10% FBS (Figure 1 F). The in vitro release profiles indicated that EBN would be stable in blood circulation and responsively release the cytotoxic epirubicin in intracellular acidic environments, which could be beneficial to exerting the therapeutic effects.

### 2.2 In Vitro Accessibility to CSCs and Therapeutic Effects

**Figure 2:**
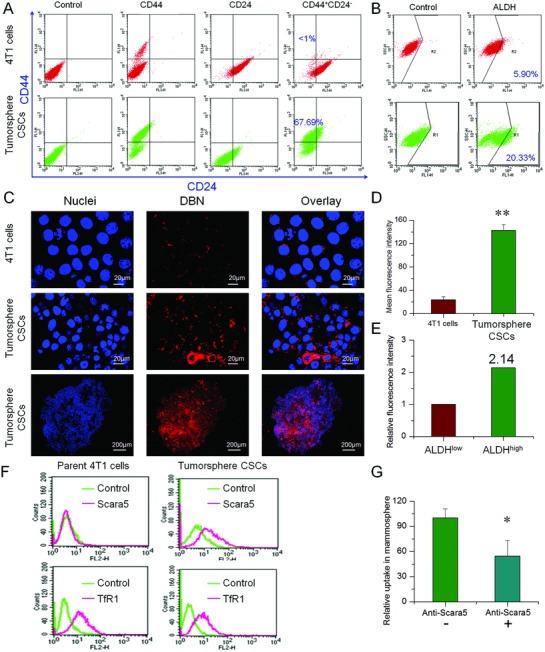
In vitro preferential accessibility to CSCs in 3D tumorsphere. A) The expression of CD44^+^CD24^−^ markers in 3D tumorsphere and parent 4T1 cells. B) The proportion of ALDH^high^ fractions in 3D tumorsphere and parent 4T1 cells. C) The uptake of DBN in CSCs‐enriched 3D tumorsphere cells and parent 4T1 cells under LCSM. By contrast, the nuclei were stained with Hoechst 33 342 for visualization. D) The mean fluorescence intensity of DBN in CSCs‐enriched tumorsphere cells and parent 4T1 cells, **p < 0.01. E) The relative uptake of DBN in ALDH^high^ and ALDH^low^ fractions of 3D tumorsphere cells. F) The expression of Scara5 and TfR in parent 4T1 cells and CSCs‐enriched 3D tumorsphere cells. G) The quantified cellular uptake of DBN in CSCs‐enriched 3D tumorsphere cells in the presence and absence of anti‐Scara5, *p < 0.05.

The 3D tumorsphere model has been identified as a reliable platform to enrich the CSCs and to evaluate the self‐renewal capability of CSCs.^30^ The highly metastatic 4T1 breast cancer cells were used to develop the CSCs‐enriched 3D tumorsphere model, which was induced by culturing 4T1 cells in ultralow attachment plates. The CD44^+^CD24^−^ and ALDH1 markers, that have been conventionally identified as CSCs markers of breast cancer,^1^, ^2^, ^14^ were used to characterize the stemness of 4T1‐induced tumorsphere cells. The expressions of ALDH1, CD44, and CD24 in tumorsphere cells were determined by flow cytometer assays. Our data suggested that the CD44^+^CD24^−^ cells were highly detected in 67.69% of tumorsphere cells, but rarely detected in parent 4T1 cancer cells (2D cultured by default) (less than 1%) (**Figure** **2**A). Meanwhile, the percentage of ALDH^high^ cells was only 5.9% in parent 4T1 cells but obviously increased to 20.33% in tumorsphere cells (Figure 2B). Therefore, compared to the parent 4T1 cells, the typical CSCs‐like CD44^+^CD24^−^ cells or ALDH^high^ cells could be largely enriched in 3D tumorsphere models.

Preferential CSCs‐accessibility is the essential prerequisite to eradicating the CSCs for anti‐metastasis therapy. The preferential access of DBN to CSCs was evaluated in the CSCs‐enriched 3D tumorsphere model and parent 4T1 cancer cells. With regards to the enrichment of CSCs in 3D tumorsphere, the potential of CSCs‐accessibility can be expressed by the enhanced uptake in tumorsphere cells compared to that in parent 4T1 cells. The internalization of DBN in tumorsphere and parent 4T1 cells were examined using laser confocal scanning microscopy (LCSM), which displayed as red fluorescence signals in the captured images. As depicted in Figure 2C, the fluorescence signals of DBN could be extensively detected in the 3D tumorsphere cells with strong intensity, but slightly observed in parent 4T1 cells. The flow cytometry analysis showed that the fluorescence intensity of DBN in CSCs‐enriched tumorsphere cells was 5.9‐fold higher than that in parent 4T1 cells (Figure 2D), revealing the preferential accessibility of DBN to CSCs‐enriched tumorsphere cells. Moreover, the mean fluorescence intensity of DBN in ALDH^high^ fractions of tumorsphere cells was 2.14‐fold higher than that in ALDH^low^ fractions (Figure 2E; Figure S1, Supporting Information). Therefore, these results effectively verified the efficient internalization of DBN in CSCs‐enriched tumorsphere and its preferential accessibility to the ALDH^high^ CSCs fractions.

Then, we attempted to elucidate the possible mechanism for the preferential CSCs‐accessibility of DBN. Previous reports indicated that ferritin could bind to the specific receptors of TfR1 and Scara5 to facilitate their internalization into cancer cells.^25^, ^26^ We characterized the expression of these typical receptors in 4T1‐mammosphere and parent 4T1 cells by flow cytometry (Figure 2F). Our data suggested that the Scara5 receptors were largely upregulated in 3D tumorsphere cells versus parent 4T1 cancer cells, whereas the expression of TfR1 was rarely changed between them. It has been evidenced that Scara5 was the specific receptors of L‐ferritin.^25^, ^31^ Horse apoferritin was composed of 24 subunits polypeptides with nearly 92% of light (L‐) chains (22/24), which was typically regarded as L‐Ferritin.^32^ In view of the high upregulation of Scara5 receptors in tumorsphere cells over parent 4T1 cells, it was rational to envision that the Scara5 receptors would be responsible for the CSCs‐specific accessibility of DBN. To confirm this deduction, we blocked the Scara5 receptors with specific monoclonal antibody (PA5‐20 766, Invitrogen) and retested their internalization in tumorsphere cells. The fluorescence intensity of DBN in tumorsphere cells was significantly reduced by 45% upon the blockage of Scara5 receptors (Figure 2G). As a result, DBN would be preferentially internalized by CSCs‐enriched tumorsphere cells via the Scara5‐mediated pathway.

**Figure 3:**
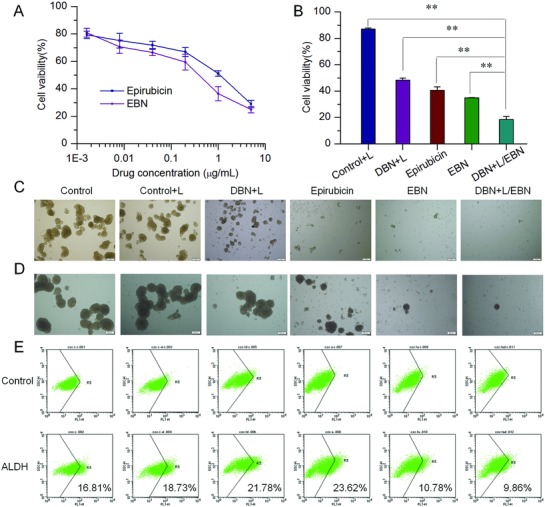
The in vitro therapeutic efficacy of DBN+L/EBN combination therapy on the viability, sphere‐forming, and ALDH^high^ CSCs fractions of tumorsphere cells. A) The inhibitory effects of EBN on the viability of parent 4T1 cells. B) The inhibitory effects of various groups on the viability of parent 4T1 cells, **p < 0.01. C) The inhibition of various groups on the tumorsphere forming ability of 4T1 cells, scale bar = 200 µm. D) The inhibition of various groups on destroying the already existing tumorspheres, scale bar = 200 µm. E) The effects on eliminating ALDH^high^ CSCs fractions in 3D tumorsphere model from each treatment.

Thereafter, the in vitro therapeutic effects of DBN and EBN were evaluated in metastatic 4T1 cancer cells. Both epirubicin and EBN presented significant inhibition on the viability of these two cell lines in a concentration‐dependent manner (**Figure** **3**A), and the average half‐inhibitory concentration (IC_50_) was 0.42 µg mL^−1^ for EBN and 1.26 µg mL^−1^ for free epirubicin. Then, cells were respectively treated with laser alone, DBN+L, epirubicin, EBN, and DBN+L/EBN to evaluate the inhibitory effects on cell viability. The DBN+L/EBN treatment resulted in an 82% inhibition of cell viability, which was significant higher than that of DBN+L or EBN (Figure 3B). Afterward, the residual cells were performed tumor‐sphere forming assays to characterize the self‐renewal capacity (Figure 3C). At day 4 after the incubation, plenty of cell‐spheres were readily detected in DBN+L group, but only small cell clusters or single cells were detected in epirubicin, EBN, and DBN+L/EBN groups, suggesting the effective inhibition on the self‐renewal ability of residual cells. In light of the efficient accessibility to CSCs, we examined the therapeutic effects on destroying already existing tumorspheres and eradicating the proportion of ALDH^high^ CSCs fractions. At 8 days of incubation after this treatment (Figure 3D; Figure S2, Supporting Information), the existing tumorsphere was not significantly changed in DBN+L group, but obviously destroyed in EBN and DBN+L/EBN groups. Moreover, the proportions of ALDH^high^ cells from each group were determined by flow cytometry after their staining with the Aldefluor fluorescent reagent (Figure 3E). When compared to the negative control, the ALDH^high^ CSCs fractions were unexpectedly increased in DBN+L and free epirubicin groups, but significantly reduced by the EBN or DBN+L/EBN treatment (Figure 3E). Similarly, the therapeutic effects of the DBN+L/EBN and EBN treatments on cell viability, inhibiting tumorsphere forming, and destroying already existing tumorspheres were also validated in human MDA‐MB‐231 cells (Figures S3 and S4, Supporting Information).

### 2.3 In Vivo Tumor Targeting, Deep Penetration, and CSCs Accessibility of DBN

**Figure 4:**
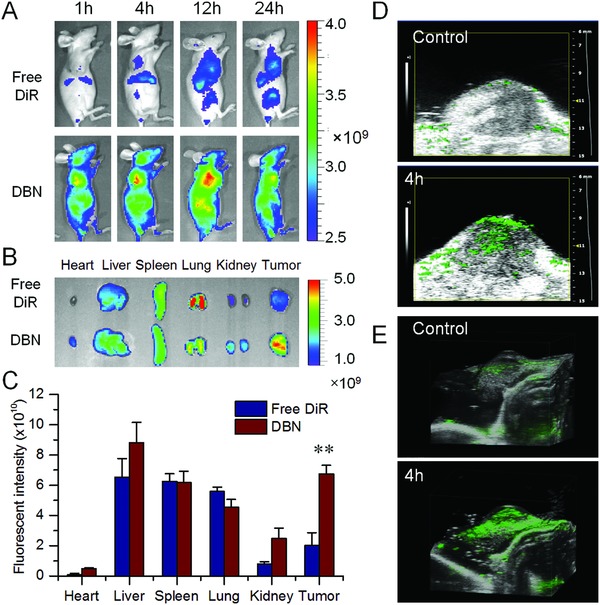
The in vivo distribution of DBN in 4T1‐induced orthotopic breast cancer model. A) The in vivo imaging of DBN and free DiR in tumor bearing mice at different time points after injection. B) The ex vivo imaging of DBN and free DiR in the major organs at 4 h of injection. C) The quantified distribution of DBN and free DiR in major organs at 4 h postinjection, **p < 0.01. D) The photoacoustic imaging of DBN in tumor at 4 h after injection. E) The reconstructed 3D distribution profiles of DBN in tumor.

The specific accumulation of DBN in tumor was examined in an orthotopic breast cancer model, which was monitored by in vivo imaging system (Spectrum, Perkin‐Elmer, USA). DBN and free DiR were respectively injected to the tumor bearing mice via tail vein for the imaging. In **Figure** **4**A, the fluorescence signals of DBN were obviously detected in the tumor sites at 1 h after injection and gradually increased with time. The signals of DBN peaked at 12 h and maintained at a high level even at 24 h after injection, which was significantly higher than that of free DiR. The whole body distribution of DBN in mice could be owing to the long blood circulating ability of the biomimetic nanocages. The ex vivo images indicated that the fluorescent signals of DBN were largely detected in liver, tumor, and lung (Figure 4B). Typically in tumor, the DBN treatment resulted in a 3.4‐fold enhancement of tumor accumulation than free DiR (Figure 4C). Moreover, the penetration of DBN in tumor mass was determined by photoacoustic imaging system (Vevo 2100 LAZR, VisualSonic FujiFilm), wherein DBN was denoted as green signals in the captured images. As shown in Figure 4D and Figure S5 (Supporting Information), DBN could be extensively detected in the exterior and interior sides of tumor mass. In the reconstructed 3D profiles, the extensive distribution of DBN in whole tumor mass was feasibly validated (Figure 4E). The measured results effectively verified the superior tumor targeting and deep penetrating capabilities of DBN in the orthotopic metastatic breast cancer model, which could mainly be ascribed to innate tumor‐targeting potential of the biomimetic nanocages and the small particle size of the DBN system. Conceivably, the efficient tumor accumulation and penetration capacity of DBN would significantly increase the possibility of DBN to be exposed to CSCs in tumor mass.

**Figure 5:**
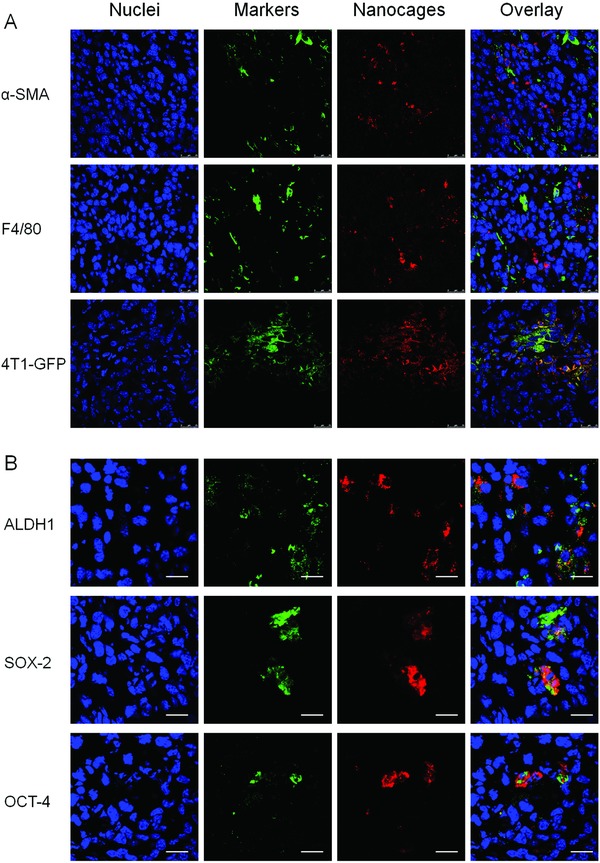
The in vivo cellular internalization and preferential CSCs‐accessibility in tumor sites. A) The in vivo internalization of DBN by various cells of CAF, TAM, and 4T1‐GFP cancer cells at tumor sites. By contrast, the nuclei were stained with DAPI for visualization, scale bar = 25 µm. B) The in vivo preferential CSCs‐accessibility of nanocages, wherein CSCs were denoted as cells with high expression of ALDH, SOX‐2, and OCT‐4 markers in tumor mass, scale bar = 20 µm.

CSCs are the rare sub‐population of cancer cells in the heterogeneous tumor tissues.^2^, ^14^ Recently, it was reported that the ALDH^high^ CSCs in hypoxic niche were more proliferative and located more centrally in the tumor mass.^33^ Accordingly, the improved tumor accumulation and deep penetration of DBN in tumor mass would rationally increase their accessibility to the CSCs. To distinguish the accessibility to CSCs in tumor, the in vivo internalization of DBN by various cells in tumor mass was determined by LCSM. Besides the initial insults of cancer cells in tumor mass, CAF and TAM are the most abundant accessary cells limiting the accessibility of therapeutic agents to cancer cells and CSCs in tumor regions.^4^, ^10^, ^34^ To distinguish their internalization into specific cells in tumor mass, the sections were stained with specific antibodies of α‐smooth muscle actin (α‐SMA) and F4/80 to outline the phenotype of CAF and TAM in tumor regions (green signals). As shown in **Figure** **5**A, DBN could be effectively internalized by some but not all cells in tumor regions. The red signals of DBN were barely colocalized with the green signals of α‐SMA and slightly merged with those of F4/80, suggesting their limited internalization into CAF and TAM in tumor regions. Moreover, to clarify the specific accessibility to cancer cells, the colocalization of DBN with 4T1 cancer cells with stable expression of green fluorescence protein (4T1‐GFP) was measured. In the captured mages, the red signals of DBN were largely distributed in the regions of 4T1‐GFP cell clusters, indicating their specific accessibility to cancer cells in tumor mass. These observations suggested that the DBN could flexibly avoid the unexpected internalization by stromal cells and be specifically accessed to cancer cells in tumor regions.

The aforementioned results have evidenced that DBN could be largely internalized by CSCs‐enriched tumorsphere cells, especially the ALDH^high^ CSCs fractions (Figure 2). Moreover, ferritin can be specifically recruited by CSCs in vivo to facilitate the tumor progression.^26^ Based on this rationale, the highly accumulated DBN in tumor can significantly improve the specific recognition and selective internalization by CSCs. To determine the preferential accessibility to CSCs, the sections were respectively labeled with specific CSCs biomarkers of ALDH1, SOX‐2, and OCT‐4 by immunofluorescence staining (Figure 5B). In the captured images, the red fluorescence signals of nanocages were largely colocated with the green signals of various CSCs markers, which effectively suggested the selective internalization by cancer cells with high expression of CSCs markers in tumor mass. Thus, the biomimetic nanocages could be rationally considered as an encouraging nanoplatform to promote the preferential drug accessibility to CSCs in tumor.

### 2.4 In Vivo Tumor Penetration and CSCs Accessibility of EBN after DBN+L Treatment

**Figure 6:**
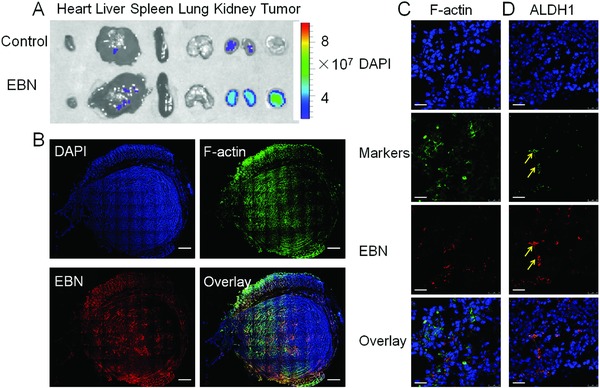
The in vivo distribution, deep penetration, and CSCs‐accessibility of EBN in tumor mass after DBN+L treatment. A) The ex vivo distribution of EBN by recording the fluorescence signals of epirubicin. B) The permeation of EBN in the whole tumor mass, wherein the actin and nuclei were respectively stained with phalloidin‐FITC and DAPI for visualization under LCSM, scale bar = 1.0 mm. C) The enlarged in vivo cellular uptake of EBN in tumor sites, scale bar = 25 µm. D) The in vivo accessibility of EBN to ALDH^high^ CSCs cells in tumor sites, which was denoted as yellow arrows, scale bar = 25 µm.

To facilitate the combinational therapy of DBN‐mediated photothermal effects and chemotherapeutic effects of EBN, EBN was given to the tumor bearing mice after laser irradiation. The fluorescence signals of EBN were detected using the imaging system and LCSM to monitor their tumor accumulation, penetration, and specific accessibility to ALDH^high^ CSCs in tumor (**Figure** **6**). The epirubicin alone could be used as a fluorescence probe to detect the in vivo localization of EBN in tumor regions, which would not be interfered with the fluorescence signal of DBN. In the ex vivo profiles, the fluorescence signals of EBN were mainly observed in kidney and tumor, but minimally detected in other organs, suggesting their superior accumulation in tumor mass (Figure 6A). Afterward, the tumor mass was sectioned and the fluorescence signals of EBN were recorded using LCSM. By contrast, the nuclei and F‐actin were counterstained as control. In the whole tumor profiles, the red fluorescence signals of EBN could be extensively detected with high intensity, wherever in the exterior tumor regions or in the interior zones of tumor mass (Figure 6B). In the enlarged profiles, the cellular internalization of EBN could be readily visualized (Figure 6C). Moreover, the red signals of EBN could be considerably colocalized with the ALDH1 markers of CSCs, suggesting the efficient in vivo accessibility of EBN to CSCs in tumor (Figure 6D). As a result, EBN could flexibly permeate throughout the whole tumor mass and be effectively assessed to the ALDH^high^ CSCs fractions in tumor, thereby holding great premise to eradicate tumor CSCs for anti‐metastasis therapy.

### 2.5 In Vivo Therapeutic Efficacy on Tumor Progression and Lung Metastasis

**Figure 7:**
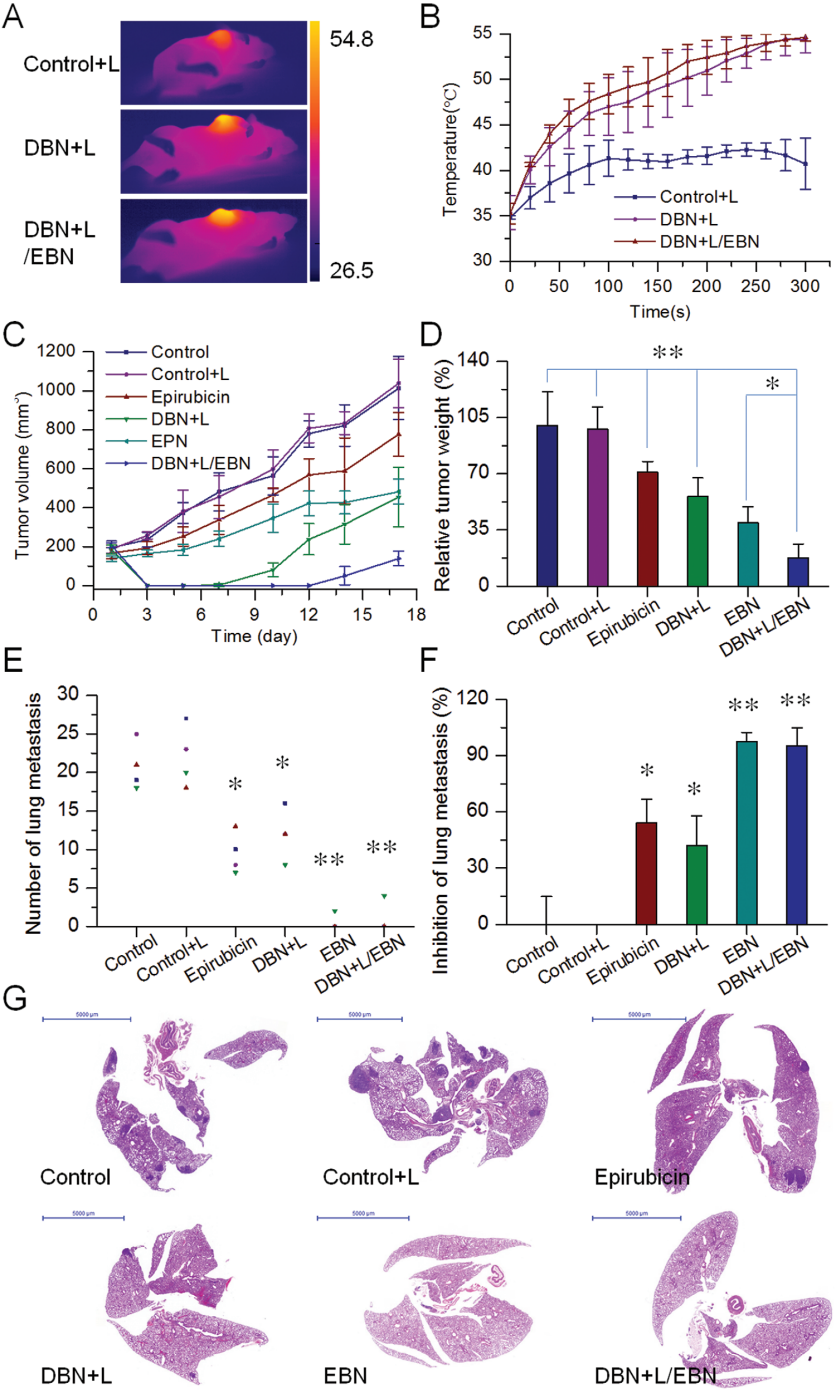
The in vivo therapeutic effects on tumor growth and lung metastasis of metastatic breast cancer model. A) The typical thermal images of tumor bearing mice. B) The temperature changes in tumor from each laser‐irradiated group. C) The tumor growth profiles from each group. D) The relative tumor weight from each group, *p < 0.05, **p < 0.01. E) The number of lung metastatic nodules from each group, *p < 0.05, **p < 0.01. F) The inhibition on the incidence of lung metastasis from each treatment, *p < 0.05, **p < 0.01. G) The histological examination of lung tissues from each group, scale bar = 5000 µm.

CSCs have been evidenced as the roots of tumor initiation and metastasis.^2^, ^7^ To determine the merit of the preferential accessibility of DBN and EBN to tumor CSCs, the in vivo therapeutic effects were measured in an orthotopic metastatic breast cancer model. In our previous results and preliminary experiments, we have found that the free DiR plus laser irradiation and DBN without laser irradiation had no inhibitory effects on tumor growth and lung metastasis of breast cancer (Figure S6, Supporting Information).^28^ In this study, the tumor‐bearing mice were respectively treated with saline control, laser alone, DBN+L, free epirubicin, EBN, and DBN+L/EBN at 1.25 mg kg^−1^ of DiR and/or 5 mg kg^−1^ of epirubicin. Upon NIR laser irradiation, the surface temperature at tumor sites in DBN+L and DBN+L/EBN could be significantly increased over 52 °C, which was much higher than that in laser alone (**Figure** **7**A,B). In the tumor growth profiles (Figure 7C), free epirubicin and EBN treatment showed a moderate inhibition on tumor growth with the inhibitory rates of 29.1% and 60.4% versus saline control, respectively. The single treatment of DBN+L resulted in a notable shrinkage of tumor size within the first 4 days, but then unexpectedly presented an obvious tumor regrowth thereafter, ultimately leading to a 44.1% reduction of tumor growth. By contrast, the laser alone had no inhibition on tumor growth. In particular, in the DBN+L/EBN group, the tumor size was notably shrunk at first 9 days and then showed a slight regrowth with the ultimate inhibition rate of 82.4%, which was significantly higher than other groups. The effective inhibition of DBN+L/EBN on tumor growth was also confirmed by measuring the tumor weight (Figure 7D; Figure S7, Supporting Information). Thereby, the DBN+L/EBN mediated combinational therapy could result in a considerable depression of tumor growth, which was much more effective than other single treatment of DBN+L or EBN.

Of note, the effectiveness of DBN+L/EBN mediated combinational therapy on suppressing tumor metastasis was also evaluated. Lungs are one of the most frequent sites of metastasis in both preclinical models and patients.^21^, ^35^ At the end time of treatment, the lung tissues from each group were collected and the visually detected metastatic nodules in each lung were recorded to evaluate the therapeutic effects on suppressing the incidence of lung metastasis (Figure 7E–G; Figure S8, Supporting Information). Compared to the saline control, the DBN+L treatment showed a 42.2% inhibition on the incidence of lung metastasis while the laser alone had no inhibitory effects. By contrast, the free epirubicin treatment depicted a moderate suppression of lung metastasis with the inhibitor rate of 54.2%. Particularly in EBN or DBN+L/EBN group, the incidence of lung metastasis was only observed in only 1 of 4 mice. The average number of lung metastasis was only about 0.5 ± 1 in EBN group and 1 ± 2 in DBN+L/EBN group, which respectively produced a 97.6% and 95.2% inhibition of lung metastasis. The efficient suppression of lung metastasis was also verified by histological examinations using the hematoxylin and eosin (H&E) staining kit (Figure 7G), in which the metastatic lesions were denoted as cell clusters of darkly stained nuclei. In the whole images of lung tissue, the metastatic foci were barely detected in EBN and DBN+L/EBN groups, but evidently observed in other groups. In addition, the good compatibility of these treatments was determined by histological examinations and detections of body weight variations (Figures S9 and S10, Supporting Information). Therefore, the EBN and DBN+L/EBN treatments depicted remarkable inhibition on the incidence of lung metastasis.

**Figure 8:**
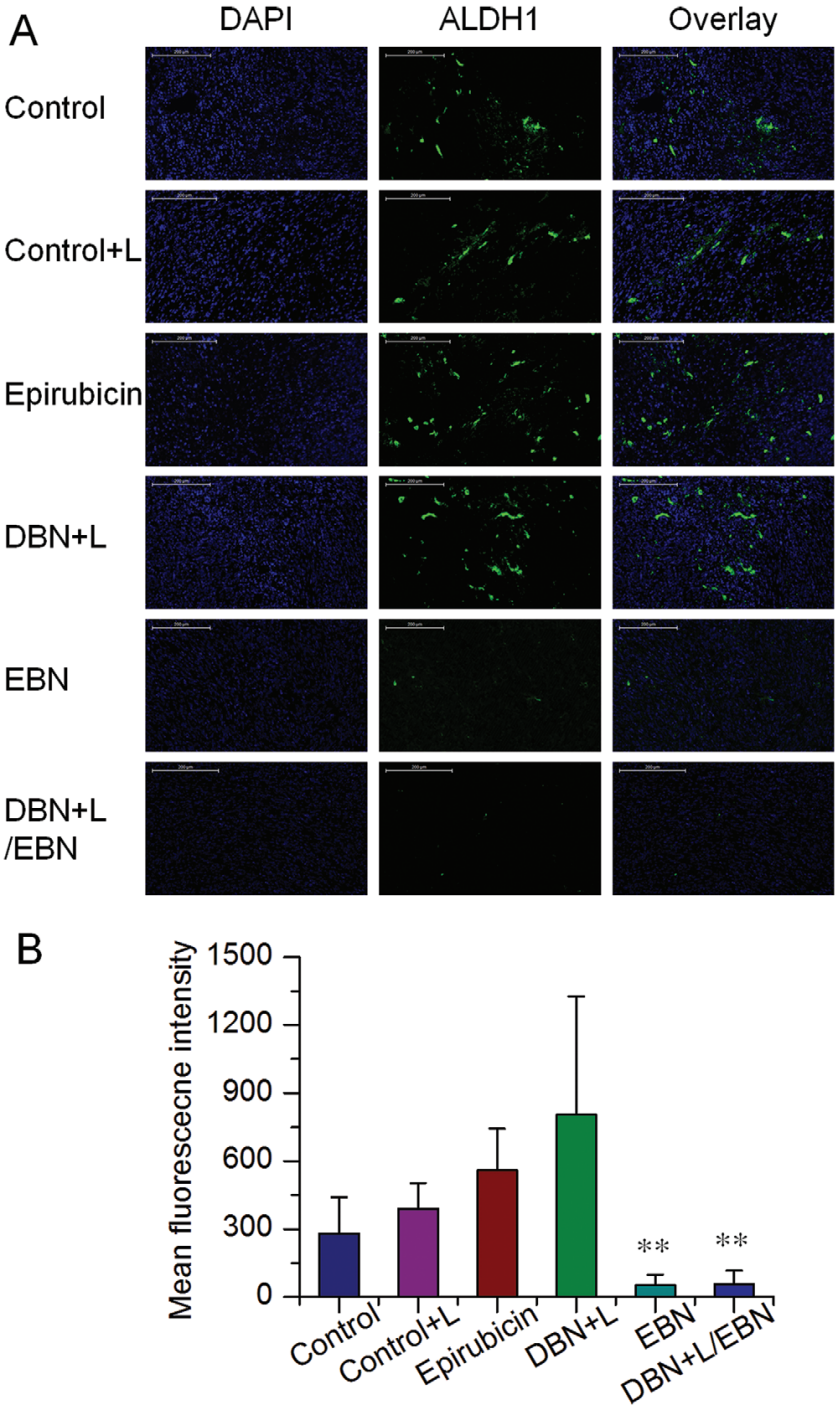
The in vivo suppression on the proportion of ALDH^high^ CSCs in tumor mass. A) Expression of ALDH^high^ markers in tumor mass from each group, which was denoted as green fluorescence signals in the captured images, scale bar = 200 µm. B) The quantified results of ALDH^high^ CSCs expressions in tumor from each group, **p < 0.01.

In view of the crucial role of CSCs fractions in tumor metastasis, the CSCs‐eliminating effects of these treatments were evaluated. The proportions of ALDH^high^ CSCs in tumor mass from each group were determined by immunofluorescence assays, which were denoted as green fluorescence signals in the captured images. The fractions of ALDH^high^ CSCs were unexpectedly increased in free epirubicin and DBN+L treatment, but remained at a low level in EBN and DBN+L/EBN groups (**Figure** **8**A). Compared to the negative control, the population of ALDH^high^ CSCs in tumor mass was obviously increased 2.0‐ and 2.9‐folds in free epirubicin and DBN+L group, indicating the enrichment of ALDH^high^ CSCs fractions in tumor mass after these treatments (Figure 8B). However, the EBN and DBN+L/EBN treatments produced 81.5% and 80.0% reduction of ALDH^high^ CSCs fractions in tumor, respectively, suggesting their considerable elimination on ALDH^high^ CSCs fractions in tumor. In addition, no significant difference was detected between EBN and DBN+L/EBN treatments. As a result, the ALDH^high^ CSCs fractions in tumors could be significantly enriched by the DBN+L mediated photothermal treatment or free epirubicin‐mediated chemotherapy, but drastically eradicated by the EBN and DBN+L/EBN treatments, which could be largely ascribed to the biomimetic nanocages mediated remarkable augment of CSCs‐accessibility. CSCs are more resistant to the damages of temperature, radiation therapy, and chemotherapeutic agents than normal cancer cells.^36^ These therapeutic modalities can cause extensive death of normal cancer cells, but are usually invalid when eliminating the CSCs, thereby unexpectedly leading to the enrichment of CSCs in tumor mass.^2^, ^37^ The DBN+L and free epirubicin treatments led to significant increase of CSCs fractions in tumor and were incompetent to prevent distant metastasis in lungs. However, in the DBN+L/EBN and EBN groups, the treatments eradicated over 80% of CSCs fractions in tumor versus negative control and resulted in efficient inhibition of lung metastasis. Thereby, the considerable elimination of ALDH^high^ CSCs fractions in tumor would play a crucial role for their efficient anti‐metastasis therapy.

## 3 Conclusions

In summary, we successfully developed a deep tumor‐penetrated biomimetic nanocages loading photothermal agent of DiR and chemotherapeutic epirubicin to improve their accessibility to CSCs for effective photo‐chemotherapy of breast cancer metastasis. The biomimetic nanocages of DBN and EBN depicted superior accumulation in tumor and flexible permeation throughout the tumor mass. Moreover, they can be largely internalized by cancer cells and preferentially accessed to ALDH^high^ CSCs fractions, but less taken up by stromal cells of CAF and TAM in tumor regions. Of note, the CSCs‐assessing nanocages of EBN and DBN+L/EBN produced considerable inhibition of primary tumor growth with notable eradication of ALDH^high^ CSCs in tumor mass, and resulted in remarkable inhibition of lung metastasis. Thus, the deep tumor penetrated biomimetic nanocages can provide an encouraging nanoplatform to ameliorate their accessibility to CSCs for effective anti‐metastasis therapy.

## 4 Experimental Sections

*Materials*: Apoferritin was provided by Sigma‐Aldrich (Shanghai, China). The NIR dye of DiR iodide was supplied by AAT Bioquest Inc (Sunnyvale, USA). The cytotoxic epirubicin and cyanine5 (Cy5) NHS ester were provided by Dalian Meilun Biological Co Ltd. (Liaoning, China). Other reagents were of analytic grade provided by Sinopharm Chemical Reagent Co. Ltd (Shanghai, China). Water was prepared from the Milli‐Q water purification system (Millipore, USA).

*Preparation and Characterization of DBN and EBN*: The loading of DiR or epirubicin into nanocages was prepared by a disassembly–assembly process using the pH changes of the solution from 2 to 7.4. In brief, apoferritin (10 mg mL^−1^) broken down into subunits by adjusting the pH to 2 with hydrochloric acid (HCl, 1 m). For preparing DBN, 120 µg of DiR in 12 µL methanol was mixed with 1 mL of apoferritin solution (10 mg mL^−1^) and incubated at room temperature for 15 min. Then, the pH of the mixed solution was turned back to pH 7.4 with sodium hydroxide solution (NaOH, 1 m) and agitated at room temperature for 2 h. Afterward, the DBN solution was purified using AKTA Purifier UPC10 with a desalting column (HiTrap, 5 mL) to remove the unloaded free DiR, and concentrated using an ultrafiltration method. The DiR amount in DBN was determined with a fluorescence spectrophotometer (Thermo Fisher Scientific Oy Ratastie2, FI‐0 1620 Vantaa, Finland). For preparing EBN, 2 mg of epirubicin in 100 µL deionized water was mixed with 1 mL of apoferritin solution (10 mg mL^−1^) and prepared as described above. Afterward, the epirubicin concentration in EBN was measured on a microplate reader (Enspire, Perkin‐Elmer, Singapore) for subsequent calculations.

The morphology of DBN and EBN was measured under the TEM (Tecnai G2 Spirit Biotwin, FEI) after negative staining with uranyl acetate. Meanwhile, the particle size distributions of DBN and EBN were measured by DLS analysis on a Malvern Zetasizer Nano ZS 90 instrument (Malvern, UK).

The in vitro heat‐generating ability of DBN was determined using an infrared thermal camera (A150‐15‐M, Irtech Ltd). In brief, 200 µL of water, free DiR in water, and DBN (100 µg mL^−1^ of DiR) were exposed to an 808 nm laser irradiation at 2.0 W cm^−2^ for 3 min. The thermal images and the temperature changes were recorded.

To evaluate the in vitro release profiles of epirubicin from EBN in physiological environments, the release behavior was respectively determined in PBS at different pH values or in PBS with 10% of FBS. In brief, 200 µL of EBN was sealed in dialysis bags and then respectively incubated with 5.0 mL of PBS at pH values of 7.4, 6.3, 5.5, and 4.7, or with PBS (pH 7.4) containing 10% FBS at 37 °C. At predetermined time intervals, the drug amount in the dissolution media was determined using the microplate reader for quantification.

*Formation and Characterization of 4T1 Induced 3D Tumorsphere*: The murine 4T1 and human MDA‐MB‐231 breast cancer cells were provided by Shanghai Cell Bank, Chinese Academy of Sciences. The 4T1 cells were cultured in the default RPMI 1640 media with 10% FBS (Gibco), and kept at 37 °C and 5% CO_2_ in a humidified incubator. The 3D tumorsphere was developed by culturing metastatic 4T1 cells in 6‐well ultralow attachment plates (40 000 cells per well) with serum free Dulbecco's modified eagle medium (DMEM)/F12 culture media supplemented with 5 µg mL^−1^ of insulin, 20 ng mL^−1^ of epidermal growth factor, 20 ng mL^−1^ of basic fibroblast growth factor, 1 × B27 (Invitrogen, USA), 0.4% (w/v) bovine serum albumin, 100 U mL^−1^ of penicillin, and 100 µg mL^−1^ of streptomycin. After 5 days of culture, the 3D tumorsphere model could be formed and used for further detections. Likewise, MDA‐MB‐231cells were cultured in DMEM with 10% FBS and cultured as described above for in vitro assessments. The 3D tumorspheres from MDA‐MB‐231 cells were developed by the aforementioned methods.

To characterize the stemness of 4T1‐induced 3D tumorspheres, they were collected and enzymatically dissociated with trypsin at 37 °C for 15 min to form single tumorsphere cells. The expressions of typical CSCs markers including CD44, CD24, and ALDH were determined by flow cytometry analysis (FACSCalibur system, BD, USA). Cells were stained with primary antibody of CD44 (701 406, Invitrogen, USA) and CD24 (14‐0242‐82, Invitrogen, USA), followed with incubation with secondary antibody of Alexa Fluor 488 labeled goat anti‐rabbit IgG (H+L) and Cyanine3 (Cy3) labeled goat anti‐rat IgG (H+L) (Beyotime, Jiangsu, China) for the analysis. Likewise, the dissociated tumorsphere cells were also stained with Aldefluor fluorescent reagent to detect the proportion of ALDH^high^ fractions. By contrast, the expressions of CD44, CD24, and ALDH markers in parent 4T1 cells were determined.

*In Vitro Preferential Accessibility to CSCs Fractions*: To determine the CSCs‐accessibility of DBN, the internalization of DBN by CSCs‐enriched 3D tumorsphere model and parent 4T1 cancer cells was evaluated using LCSM (Olympus, Japan) and flow cytometry (FACSCalibur, BD). DBN was incubated with 3D tumorsphere cells or 4T1 cells for 4 h for the measurements. As control, the nuclei were stained with Hoechst 33 342 (Beyotime, Jiangsu, China). The uptake of DBN in 3D tumorsphere cells and parent 4T1 cells was visualized under LCSM, which was denoted as red fluorescence signals in the captured images. Moreover, the internalization was quantified by flow cytometer analysis.

Then, the preferential accessibility of DBN to ALDH^high^ CSCs fractions in 3D tumorsphere was determined. DBN was incubated with 3D tumorspheres for 4 h and dissociated into single tumorsphere cells with trypsin as described above. After staining with the Aldefluor fluorescent reagent, the fluorescence signals in ALDH^high^ CSCs or ALDH^low^ cells were determined by flow cytometry.

*Possible Mechanism for the Preferential CSCs‐Accessibility of DBN*: To elucidate the mechanism for the preferential CSCs‐accessibility of DBN, the specific expressions of TfR1 and Scara5 receptors in 3D tumorsphere cells and parent 4T1 cells were characterized by flow cytometry. To confirm the expressions of TfR1 and Scara5 receptors, the tumorsphere cells and parent 4T1 cells were respectively treated with primary antibody against TfR (ab60344, Abcam, UK) and Scara5 (Invitrogen, USA), and respectively incubated with the secondary antibody of Cy3‐labeled goat anti‐rat IgG (H+L) and Alexa Fluor 488 labeled goat anti‐rabbit IgG (H+L) (Beyotime, Jiangsu, China). Cells without any staining were performed as negative control. Afterward, the expressions of TfR1 and Scara5 in tumorsphere cells and parent 4T1 cells were analyzed by flow cytometry (FACSCalibur, BD, USA).

To detect whether Scara5 was responsible for the preferential CSCs‐accessibility of DBN, the Scara5 receptors were masked in 3D tumorsphere using specific antibody against Scara5 (Invitrogen, USA), and the internalization in the CSCs‐enriched tumorsphere model was retested. In brief, the 3D tumorspheres in a 6‐well plate were pretreated with the specific antibody of Scara5 for 1 h and then incubated with DBN for 4 h. By contrast, the untreated 3D tumorspheres were incubated with DBN for 4 h as control. Afterward, the fluorescence intensities of DBN in these two groups were quantified by flow cytometry analysis.

*Cytotoxicity in 4T1 Cells*: The cytotoxicity of EBN and free epirubicin was evaluated in parent 4T1 cells. Cells were seeded into normal 96‐well culture plate at 4000 cells per well and incubated in the RPMI 1640 media overnight for the attachment. Then, free epirubicin and EBN were added to each well at concentrations ranging from 1.6 ng mL^−1^ to 5 µg mL^−1^ and incubated for further 24 h. Thereafter, the cell viability in each group was analyzed by 3‐(4,5‐dimethylthiazol‐2‐yl)‐2,5‐diphenyltetrazolium bromide (MTT) assays (Enspire, Perkin‐Elmer, Singapore). For the cytotoxicity in MDA‐MB‐231 cells, cells were seeded into 96 well pate at 5000 cells per well and cultured overnight, and then incubated with EBN or free epirubicin with the concentration within 3.2 ng mL^−1^ to 10 µg mL^−1^. After 24 h incubation, the cell viability was determined using MTT assays. Cells without any treatment were performed as negative control.

Then, to determine the combinational effects of DBN plus laser irradiation and EBN on cell viability, the seeded cells in 96‐well culture plate (4000 cells per well) were respectively treated with laser alone, DBN+L, free epirubicin, EBN, and DBN+L/EBN at 0.25 µg mL^−1^ of DiR or 1 µg mL^−1^ of epirubicin. For laser irradiated groups, cells were exposed to the 808 nm laser at 2.0 W for 3 min. After 24 h of incubation, the cell viability in each group was measured by MTT assays. Cells without any treatment were performed as negative control. In MDA‐MB‐231 cells, the seeded cells in 96‐well plate were respectively treated with control, Control+L, DiR+L/epirubicin, DBN+L, epirubicin, EBN, DBN/EBN, and DBN+L/EBN at 0.25 µg mL^−1^ of DiR or 1 µg mL^−1^ of epirubicin. After 24 h of incubation, the cell viability in each group was determined by MTT assays.

Thereafter, to evaluate their effects on self‐renewal capacity of cancer cells, these residual cells from each group were seeded to 6‐well ultralow attachment plate at 40 000 cells per well, and cultured in the serum free culture media for 4 days. The formation of 3D tumorsphere from each treatment was monitored and imaged under the inverted microscope. Cells without any treatment were performed as negative control. In MDA‐MB‐231 cells, the residual cells from each group were seeded to 6‐well ultralow attachment plate at 120 000 cells per well, cultured in the serum free media and then measured as described above.

*Eliminating ALDH^high^ CSCs in Tumorsphere*: The effects of the combinational therapy on destroying existing tumorsphere and eliminating ALDH^high^ CSCs were measured using the CSCs‐enriched 3D tumorsphere models, which were developed from 40 000 cells per well in ultralow attachment 6‐well plates (Figure S2, Supporting Information). The 3D tumorsphere models were respectively treated with laser alone, DBN+L, free epirubicin, EBN, and DBN+L/EBN at 0.25 µg mL^−1^ of DiR or 1 µg mL^−1^ of epirubicin. For laser irradiated groups, cells were exposed to the 808 nm laser at 2.0 W for 4 min. After 8 days of incubation, the morphology of the tumorsphere from each treatment was monitored and imaged under the inverted microscope. Cells without any treatment were performed as negative control. For MDA‐MB‐231 cells, the existing 3D tumorspheres were respectively treated with control, Control+L, DiR+L/epirubicin, DBN+L, epirubicin, EBN, DBN/EBN, and DBN+L/EBN at 0.25 µg mL^−1^ of DiR or 1 µg mL^−1^ of epirubicin, and then monitored as described above. Afterward, the tumorspheres from each group were dissociated into single cells, stained with the Aldefluor fluorescent reagent, and analyzed using the flow cytometer system (FACSCalibur, BD, USA) to determine the proportion of ALDH^high^ cells in each group.

*In Vivo Tumor Accumulation and Deep Tumor Penetration of DBN in Metastatic Breast Cancer Model*: Female nude mice (18–22 g, BALB/c) were ordered from Shanghai Experimental Animal Center, Chinese Academy of Sciences (Shanghai, China) and used to induce the orthotopic breast cancer model, which was developed by subcutaneous injection of 4T1 cells to the mammary pad of nude mice at 1 × 10^6^ cells per mouse. The experiments were performed according to the protocols approved by the Institutional Animal Care and Use Committee of Shanghai Institute of Materia Medica, Chinese Academy of Sciences.

When the tumor volume reached about 100–150 mm^3^, DBN and free DiR solutions were respectively given to the tumor bearing mice via tail injection. At predetermined time points after injection, mice were anesthetized and the fluorescence signals at tumor sites were recorded using the in vivo imaging system (IVIS Spectrum, PerkinElmer). At 4 h, the major organs including heart, liver, spleen, lung, kidney, and tumor tissues were carefully removed and analyzed using the imaging system. The intensity of fluorescence signals in each organ was recorded for quantification.

Then, the deep penetration of DBN in tumor was measured by photoacoustic imaging (Vevo 2100 LAZR, VisualSonic FUJIFILM). DBN was injected to the tumor bearing mice via tail vein. At 2, 4, and 8 h after injection, mice were anesthetized and the tumor tissues were scanned using the photoacoustic imaging system, wherein the signals of DBN were denoted as green fluorescence spots. Typically at 4 h, the 3D profiles of DBN in tumor mass were scanned and reconstructed by the accompanied software to clarify the penetration in tumor mass. The photoacoustic signals of tumor prior to the injection were recorded as negative control and the ultrasound signals were documented to provide the anatomical images of tumor mass.

*In Vivo CSCs Accessibility in Metastatic Breast Cancer Model*: The in vivo cellular uptake of DBN by various cells in tumor sites were determined using LCSM. At 4.0 h injection of DBN, the tumor tissues were carefully removed and embedded into tissues freezing media for cryostat section at 10 µm (CM1950, Leica). To clarify the internalization by various cells in tumor sites, the sections were respectively stained with specific antibodies of α‐SMA (Abcam, ab5694), F4/80 (Abcam, ab6640), and then incubated with the secondary antibody of Alexa Fluor 488 labeled IgG (H+L) (Beyotime, Jiangsu, China). By contrast, the nuclei were stained with 4′,6‐diamidino‐2‐phenylindole, dihydrochloride (DAPI) for the observations. To detect the accessibility of DBN to cancer cells, the tumor bearing mice were developed with 4T1‐GFP cancer cells and performed as described above. The tumor sections were stained with DAPI for visualization under LCSM. The uptake of DBN in cancer cells was denoted as the colocalization of red fluorescence signals with green colors of 4T1‐GFP cells.

Most importantly, to determine the preferential accessibility to CSCs fractions in tumor mass, the Cy5‐labeled nanocages were used for the detections. At 4.0 h postinjection, the tumor tissues were collected and sectioned for the measurements. The tumor sections were respectively stained with the specific antibody against the specific CSCs markers of ALDH1, SOX‐2, and OCT‐4, and then incubated with the secondary antibody of Alexa Fluor 488 labeled goat anti‐rabbit IgG(H+L) (green, Beyotime). By contrast, the sections were stained with DAPI (blue, Beyotime). In the captured images, the accessibility to CSCs was depicted as the overlay of red fluorescence signals from nanocages and the green colors of CSCs markers.

*In Vivo Deep Tumor Penetration and CSCs Accessibility of EBN in Metastatic Breast Cancer Model*: The in vivo penetration effects and CSCs‐assessing ability of EBN were also measured in the 4T1 induced metastatic breast cancer model. EBN was given to the tumor bearing mice after they were treated with DBN plus NIR laser irradiation (3.5 W cm^−2^ for 5 min). Four hours later, the tumor tissues were carefully removed and embedded into tissues freezing media for cryostat section at 10 µm (CM1950, Leica). By contrast, the sections were stained with Actin‐Tracker Green and DAPI (Beyotime, China) for visualizing under LCSM. The fluorescence signals in whole tumor area and typical regions were recorded. Moreover, to clarify the CSCs‐accessing ability of EBN in tumor sites, the tumor sections were stained with specific antibody against ALDH1, followed with the secondary antibody of Alexa Fluor 488 labeled IgG (H+L) (Beyotime, Jiangsu, China). By contrast, the nuclei were stained with DAPI for the observations. The accessibility of EBN to CSCs was denoted as the colocalization of red signals from EBN and the green colors of ALDH^high^ CSCs fractions in tumor mass.

*In Vivo Suppression of Tumor Growth, CSCs Fractions in Tumor, and Lung Metastasis of Breast Cancer*: The in vivo therapeutic effects were determined in an orthotopic metastatic breast cancer model. When the tumor volume reached 100–150 mm^3^ in volume, the tumor bearing mice were respectively treated with saline control, laser alone, DBN+L, free epirubicin, EBN, and DBN+L/EBN at 1.25 mg kg^−1^ of DiR or 5 mg kg^−1^ of epirubicin. For laser irradiated group, mice were anaesthetized and tumors were exposed to an 808 nm laser at 3.5 W cm^−2^ for 5 min for a single treatment. The thermal images and the surface temperature of tumor were monitored using the infrared thermal camera. In DBN+L and DBN+L/EBN groups, the laser irradiation was performed at 4 h after tail injection of DBN. In DBN+L/EBN group, EBN was injected to the tumor model after the laser irradiation, and then administered 5 days later for a total of 2 injections. By contrast, mice in EBN group without laser irradiation were treated at day 0 and 5 for a total 2 injections. The body weight in each group was measured and the tumor size was monitored using a digital caliper twice a week. At day 17 after the first treatment, mice were autopsied and the major organs were collected for further measurements. The tumor mass in each group was photographed and weighed to evaluate the inhibition of tumor growth. To evaluate their possible toxicity to the major organs of heart, liver, and kidney, these tissues were embedded in paraffin, sectioned at 5 µm, and then stained with H&E kit for histopathological analysis.

To determine the elimination on CSCs fractions, the tumor mass from each group were embedded in paraffin and sectioned at 5 µm. The sections were incubated with specific antibody against ALDH1 (Abcam, ab52492) and followed with incubation with the secondary antibody of Alexa Fluor 488 labeled goat anti‐rabbit IgG(H+L) (green, Beyotime). By contrast, the nuclei were stained with DAPI for the observations. Then, samples were visualized under fluorescence microscope (Nikon Eclipse TI‐SR) and the images were analyzed using the Image‐pro plus 6.0 software for quantification.

Meanwhile, the lungs from each group were collected and photographed. The visually detected metastatic nodules in each lung was carefully counted to calculate the inhibitory rate on the incidence of lung metastasis. The inhibition of lung metastasis was defined as the average number of metastatic nodules in each group comparing to that in saline control. Afterward, the lung tissues were performed histological examinations. The metastatic lesions in lung were depicted as cell clusters with darkly stained nuclei.

*Statistical Analysis*: Data were expressed as mean values ± standard deviation (SD). The statistical significance between two groups was determined by a two‐tailed Student's *t*‐test. The difference was considered as significant when the *p* value was less than 0.05.

## Conflict of Interest

The authors declare no conflict of interest.

## Supporting information

Supplementary

## References

[1] J. P. Medema , Nat. Cell Biol. 2013, 15, 338. 23548926 10.1038/ncb2717

[2] D. R. Pattabiraman , R. A. Weinberg , Nat. Rev. Drug Discovery 2014, 13, 497. 24981363 10.1038/nrd4253 PMC4234172

[3] a) K. L. Harper , M. S. Sosa , D. Entenberg , H. Hosseini , J. F. Cheung , R. Nobre , A. Avivar‐Valderas , C. Nagi , N. Girnius , R. J. Davis , E. F. Farias , J. Condeelis , C. A. Klein , J. A. Aguirre‐Ghiso , Nature 2016, 540, 588; 10.1038/nature20609 PMC5471138 27974798

[4] D. A. Lawson , N. R. Bhakta , K. Kessenbrock , K. D. Prummel , Y. Yu , K. Takai , A. Zhou , H. Eyob , S. Balakrishnan , C. Y. Wang , P. Yaswen , A. Goga , Z. Werb , Nature 2015, 526, 131. 26416748 10.1038/nature15260 PMC4648562

[5] a) R. Sun , Y. Liu , S. Y. Li , S. Shen , X. J. Du , C. F. Xu , Z. T. Cao , Y. Bao , Y. H. Zhu , Y. P. Li , X. Z. Yang , J. Wang , Biomaterials 2015, 37, 405; 25453968 10.1016/j.biomaterials.2014.10.018

[6] Y. Liu , C. Y. Chen , P. X. Qian , X. F. Lu , B. Y. Sun , X. Zhang , L. M. Wang , X. F. Gao , H. Li , Z. Y. Chen , J. L. Tang , W. J. Zhang , J. Q. Dong , R. Bai , P. E. Lobie , Q. F. Wu , S. L. Liu , H. F. Zhang , F. Zhao , M. S. Wicha , T. Zhu , Y. L. Zhao , Nat. Commun. 2015, 6, 5988. 25612916 10.1038/ncomms6988 PMC4354030

[7] Y. Z. Li , H. A. Rogoff , S. Keates , Y. Gao , S. Murikipudi , K. Mikule , D. Leggett , W. Li , A. B. Pardee , C. J. Li , Proc. Natl. Acad. Sci. USA 2015, 112, 1839. 25605917 10.1073/pnas.1424171112 PMC4330785

[8] A. Mohyeldin , T. Garzon‐Muvdi , A. Quinones‐Hinojosa , Cell Stem Cell 2010, 7, 150. 20682444 10.1016/j.stem.2010.07.007

[9] Z. Q. Zuo , K. G. Chen , X. Y. Yu , G. Zhao , S. Shen , Z. T. Cao , Y. L. Luo , Y. C. Wang , J. Wang , Biomaterials 2016, 82, 48. 26751819 10.1016/j.biomaterials.2015.12.014

[10] H. Lu , K. R. Clauser , W. L. Tam , J. Frose , X. Ye , E. N. Eaton , F. Reinhardt , V. S. Donnenberg , R. Bhargava , S. A. Carr , R. A. Weinberg , Nat. Cell Biol. 2014, 16, 1105. 25266422 10.1038/ncb3041 PMC4296514

[11] a) S. S. McAllister , R. A. Weinberg , Nat. Cell Biol. 2014, 16, 717; 25082194 10.1038/ncb3015 PMC6220424

[12] S. Shen , J. X. Xia , J. Wang , Biomaterials 2016, 74, 1. 26433488 10.1016/j.biomaterials.2015.09.037

[13] a) Y. Zhao , D. Y. Alakhova , A. V. Kabanov , Adv. Drug Delivery Rev. 2013, 65, 1763; 10.1016/j.addr.2013.09.016 PMC4174448 24120657

[14] J. E. Visvader , G. J. Lindeman , Nat. Rev. Cancer 2008, 8, 755. 18784658 10.1038/nrc2499

[15] J. L. Au , B. Z. Yeung , M. G. Wientjes , Z. Lu , M. G. Wientjes , Adv. Drug Delivery Rev. 2016, 97, 280. 10.1016/j.addr.2015.12.002 PMC4829347 26686425

[16] K. K. Ng , J. F. Lovell , G. Zheng , Acc. Chem. Res. 2011, 44, 1105. 21557543 10.1021/ar200017e PMC3196219

[17] a) L. Miao , J. M. Newby , C. M. Lin , L. Zhang , F. F. Xu , W. Y. Kim , M. G. Forest , S. K. Lai , M. I. Milowsky , S. E. Wobker , L. Huang , ACS Nano 2016, 10, 9243; 10.1021/acsnano.6b02776 PMC5515694 27666558

[18] Z. Zhang , H. Wang , T. Tan , J. Li , Z. Wang , Y. Li , Adv. Funct. Mater. 2018, 28, 1801840.

[19] a) E. Blanco , H. Shen , M. Ferrari , Nat. Biotechnol. 2015, 33, 941; 26348965 10.1038/nbt.3330 PMC4978509

[20] B. He , T. Tan , H. Wang , H. Hu , Z. Wang , J. Wang , J. Li , K. Sun , Z. Zhang , Y. Li , Adv. Funct. Mater. 2018, 28, 1705622.

[21] a) H. Q. Cao , Z. L. Dan , X. Y. He , Z. W. Zhang , H. J. Yu , Q. Yin , Y. P. Li , ACS Nano 2016, 10, 7738; 27454827 10.1021/acsnano.6b03148

[22] a) Z. Wang , P. Huang , O. Jacobson , Z. Wang , Y. Liu , L. Lin , J. Lin , N. Lu , H. Zhang , R. Tian , G. Niu , G. Liu , X. Chen , ACS Nano 2016, 10, 3453; 26871955 10.1021/acsnano.5b07521 PMC5242369

[23] a) C. Cao , X. Wang , Y. Cai , L. Sun , L. Tian , H. Wu , X. He , H. Lei , W. Liu , G. Chen , R. Zhu , Y. Pan , Adv. Mater. 2014, 26, 2566; 24532221 10.1002/adma.201304544

[24] a) X. Lin , J. Xie , G. Niu , F. Zhang , H. Gao , M. Yang , Q. Quan , M. A. Aronova , G. Zhang , S. Lee , R. Leapman , X. Chen , Nano Lett. 2011, 11, 814; 21210706 10.1021/nl104141g PMC3036786

[25] J. Y. Li , N. Paragas , R. M. Ned , A. D. Qiu , M. Viltard , T. Leete , I. R. Drexler , X. Chen , S. Sanna‐Cherchi , F. Mohammed , D. Williams , C. S. Lin , K. M. Schmidt‐Ott , N. C. Andrews , J. Barasch , Dev. Cell 2009, 16, 35. 19154717 10.1016/j.devcel.2008.12.002 PMC2652503

[26] D. L. Schonberg , T. E. Miller , Q. Wu , W. A. Flavahan , N. K. Das , J. S. Hale , C. G. Hubert , S. C. Mack , A. M. Jarrar , R. T. Karl , A. M. Rosager , A. M. Nixon , P. J. Tesar , P. Hamerlik , B. W. Kristensen , C. Horbinski , J. R. Connor , P. L. Fox , J. D. Lathia , J. N. Rich , Cancer Cell 2015, 28, 441. 26461092 10.1016/j.ccell.2015.09.002 PMC4646058

[27] X. Liu , W. Jin , E. C. Theil , Proc. Natl. Acad. Sci. USA 2003, 100, 3653. 12634425 10.1073/pnas.0636928100 PMC152977

[28] X. Y. He , X. Y. Bao , H. Q. Cao , Z. W. Zhang , Q. Yin , W. W. Gu , L. L. Chen , H. J. Yu , Y. P. Li , Adv. Funct. Mater. 2015, 25, 2831.

[29] H. Cabral , Y. Matsumoto , K. Mizuno , Q. Chen , M. Murakami , M. Kimura , Y. Terada , M. R. Kano , K. Miyazono , M. Uesaka , N. Nishiyama , K. Kataoka , Nat. Nanotechnol. 2011, 6, 815. 22020122 10.1038/nnano.2011.166

[30] B. Beck , C. Blanpain , Nat. Rev. Cancer 2013, 13, 727. 24060864 10.1038/nrc3597

[31] K. L. Fan , M. Zhou , X. Y. Yan , Protein Cell 2017, 8, 788. 28993977 10.1007/s13238-017-0481-8 PMC5676598

[32] L. Mendes‐Jorge , D. Ramos , A. Valenca , M. Lopez‐Luppo , V. M. Pires , J. Catita , V. Nacher , M. Navarro , A. Carretero , A. Rodriguez‐Baeza , J. Ruberte , PLoS One 2014, 9, e106974. 25259650 10.1371/journal.pone.0106974 PMC4178024

[33] S. L. Liu , Y. Cong , D. Wang , Y. Sun , L. Deng , Y. J. Liu , R. Martin‐Trevino , L. Shang , S. P. McDermott , M. D. Landis , S. Hong , A. Adams , R. D'Angelo , C. Ginestier , E. Charafe‐Jauffret , S. G. Clouthier , D. Birnbaum , S. T. Wong , M. Zhan , J. C. Chang , M. S. Wicha , Stem Cell Rep. 2014, 2, 78. 10.1016/j.stemcr.2013.11.009 PMC3916760 24511467

[34] a) R. Kalluri , Nat Rev Cancer 2016, 16, 582; 27550820 10.1038/nrc.2016.73

[35] a) G. Francia , W. Cruz‐Munoz , S. Man , P. Xu , R. S. Kerbel , Nat. Rev. Cancer 2011, 11, 135; 21258397 10.1038/nrc3001 PMC4540342

[36] a) M. Dean , T. Fojo , S. Bates , Nat. Rev. Cancer 2005, 5, 275; 15803154 10.1038/nrc1590

[37] a) V. P. Chauhan , R. K. Jain , Nat. Mater. 2013, 12, 958; 24150413 10.1038/nmat3792 PMC4120281

