# Supplementary material for: Deep Tumor‐Penetrated Nanocages Improve Accessibility to Cancer Stem Cells for Photothermal‐Chemotherapy of Breast Cancer Metastasis
Source: Adv Sci (Weinh). 2018 Oct 29;5(12):1801012. doi: 10.1002/advs.201801012 (PMC6299727; doi:10.1002/advs.201801012)
Supplement: Supplementary file 1 — Supplementary [file ADVS-5-1801012-s001.pdf]

Copyright WILEY-VCH Verlag GmbH & Co. KGaA, 69469 Weinheim, Germany, 2018.

ADVANCED SCIENCE Open Access

Supporting Information

for *Adv. Sci.,* DOI: 10.1002/advs.201801012

Deep Tumor-Penetrated Nanocages Improve Accessibility to Cancer Stem Cells for Photothermal-Chemotherapy of Breast Cancer Metastasis

Tao Tan, Hong Wang, Haiqiang Cao, Lijuan Zeng, Yuqi Wang, Zhiwan Wang, Jing Wang, Jie Li, Siling Wang, Zhiwen Zhang,* and Yaping Li**

Supporting Information

Deep tumor-penetrated nanocages improve accessibility to cancer stem cells for photothermal-chemotherapy of breast cancer metastasis

Tao Tan, Hong Wang, Haiqiang Cao, Lijuan Zeng, Yuqi Wang, Zhiwan Wang, Jing Wang, Jie Li, Siling Wang, Zhiwen Zhang*, Yaping Li **

Supplementary data

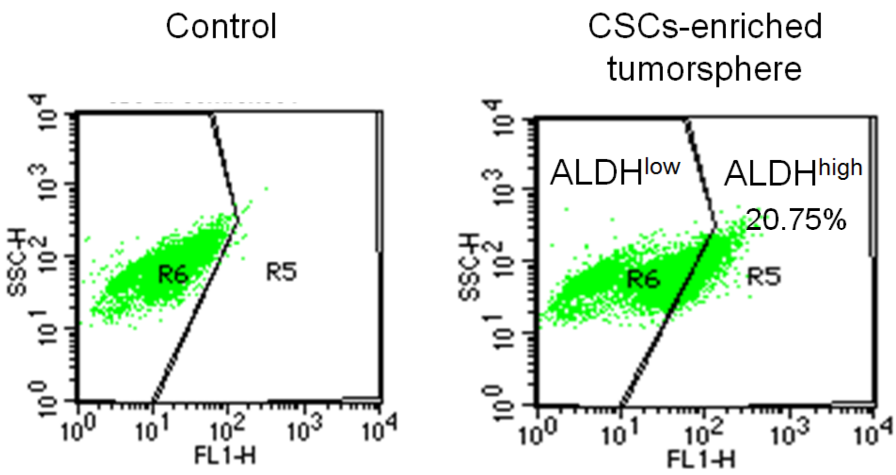

Figure S1 The analysis of ALDH^{high} and ALDH^{low} fractions in 4T1-induced CSCs-enriched tumorsphere models when comparing the internalization of DBN in these two fractions of tumorsphere cells. Cells were incubated with the Aldefluor fluorescent reagents for the measurements.

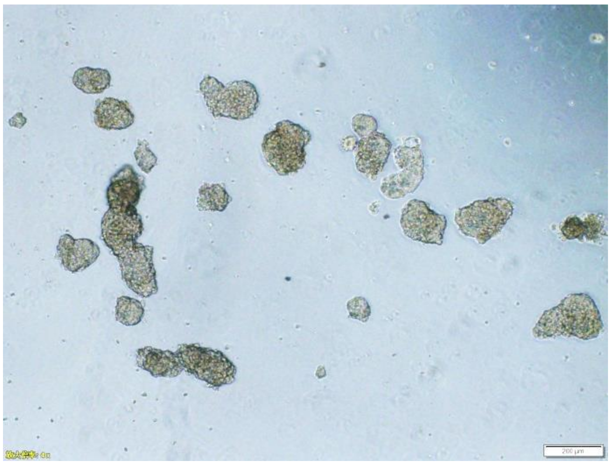

Figure S2 The typical images of 4T1 induced tumorsphere at day 0 of incubation for the tumorsphere-inhibition assays, scale bar = 200 µm.

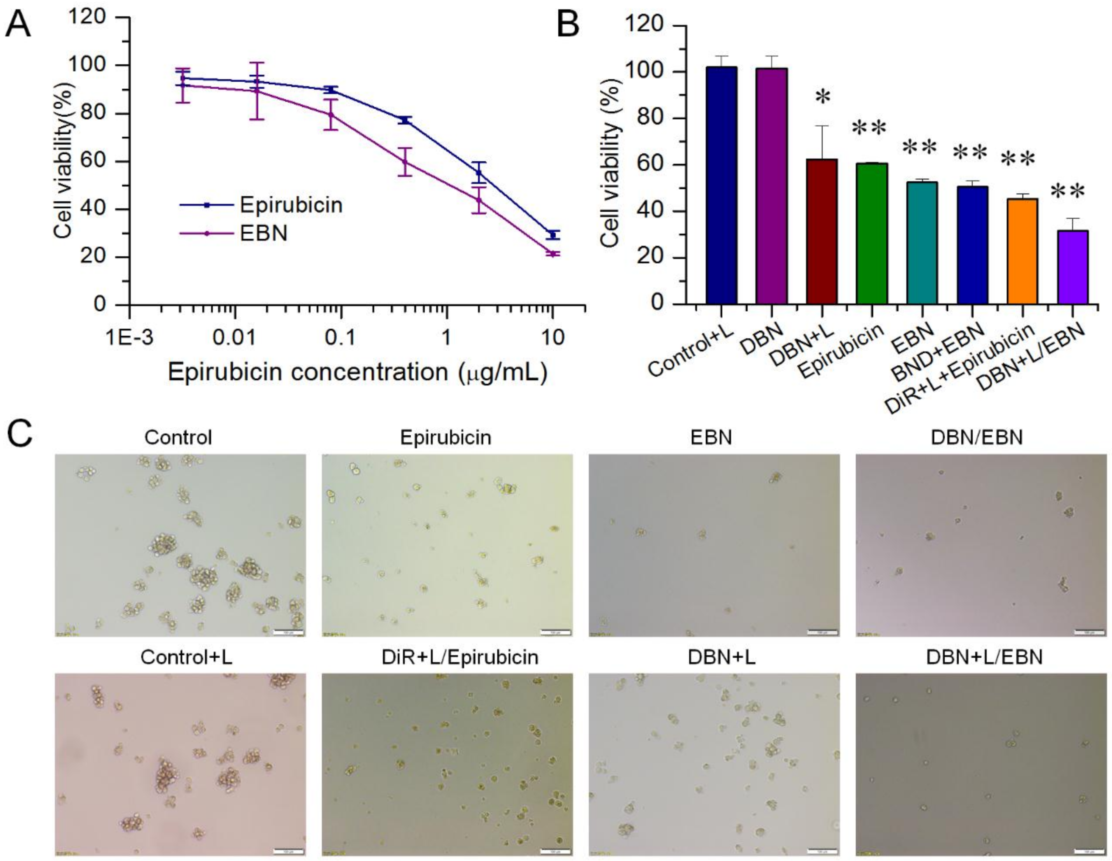

Figure S3 The *in vitro* therapeutic efficacy of DBN+L/EBN combination therapy on the viability and sphere-forming ability of MDA-MD-231 cells. For laser irradiated groups, cells were exposed to the 808 nm laser at 2.0 W for 3 min for the measurements. (A) The cytotoxicity of epirubicin and EBN in MDA-MD-231 cells; (B) The inhibitory effects of various groups on the viability of MDA-MD-231 cells at 0.25 μg/mL of DiR or 1.0 μg/mL of epirubicin. The statistical analysis was performed in comparison to the control group, * $p<0.05$, ** $p<0.01$; (C) The inhibition of various groups on the sphere-forming ability of 4T1 cells, scale bar = 100 μm.

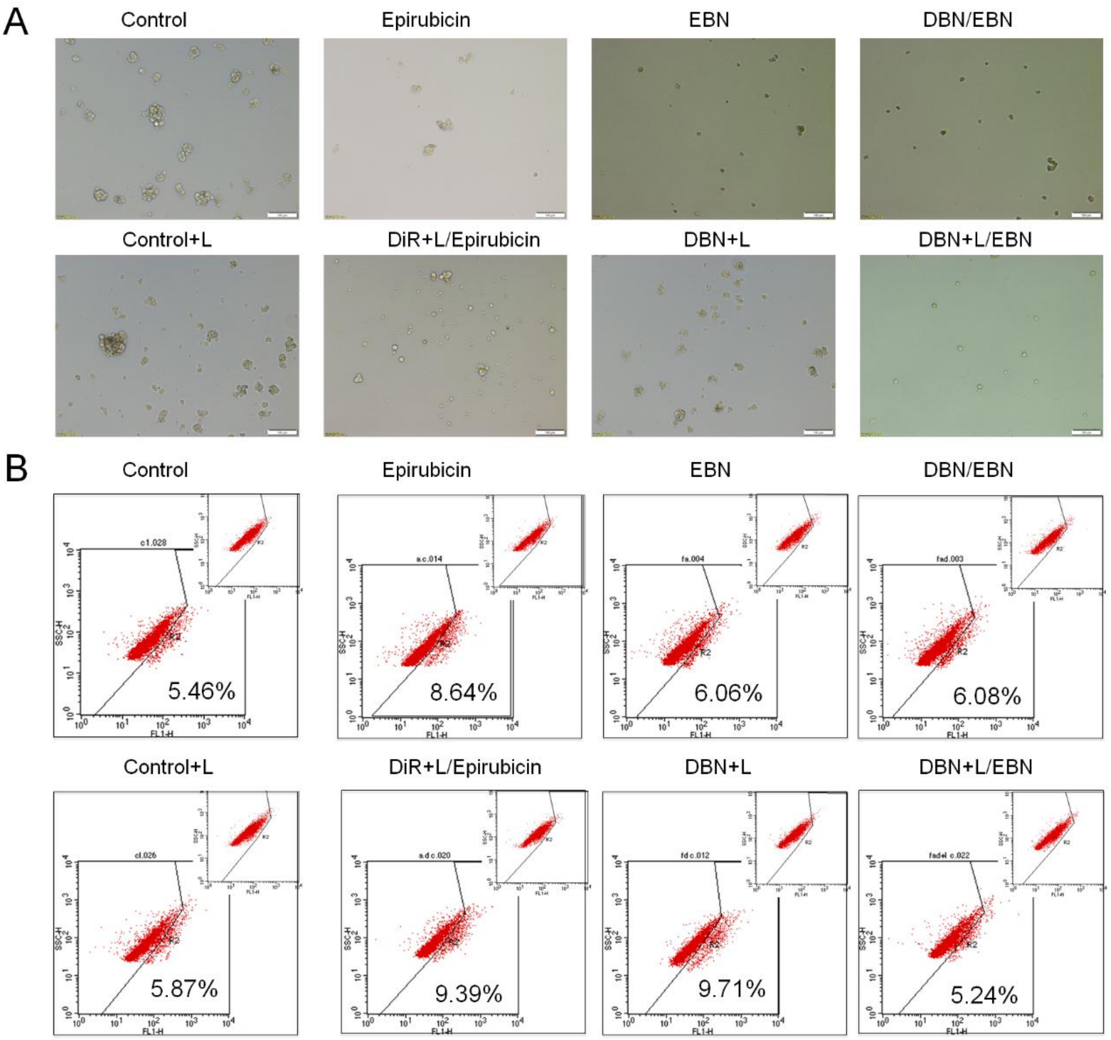

Figure S4 The therapeutic efficacy of DBN+L/EBN combination therapy on destroying the destroying the already existing tumorspheres and eliminating ALDH^{high} CSCs fractions. (A) The therapeutic effects on destroying already existing tumorspheres, scale bar = 100 µm; (B) The effects on eliminating ALDH^{high} CSCs fractions in 3D tumorsphere model from each treatment.

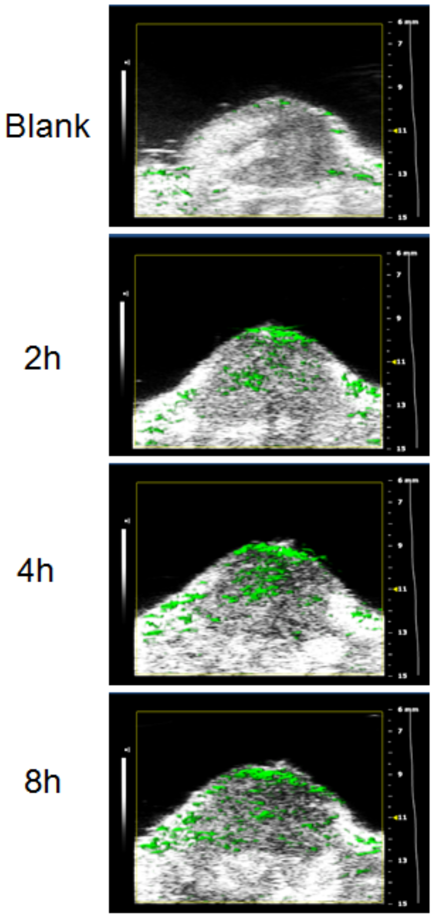

Figure S5 The photoacoustic imaging of DBN in tumor at different time points after injections, which was denoted as green fluorescent signals in the captured images.

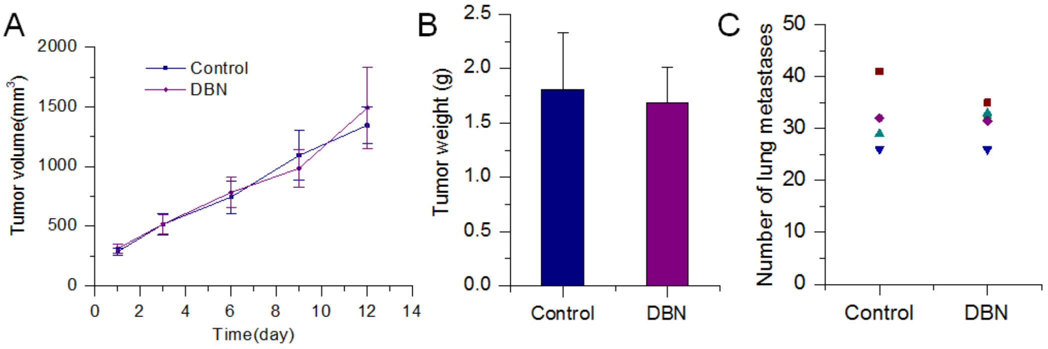

Figure S6 The therapeutic effects of DBN without laser irradiations on tumor growth and lung metastasis of breast cancer model, which confirmed the invalidity of the DBN treatment. (A) The tumor growth profiles in control and DBN groups; (B) The tumor weight in control and DBN groups; (C) The number of lung metastatic nodules in control and DBN groups.

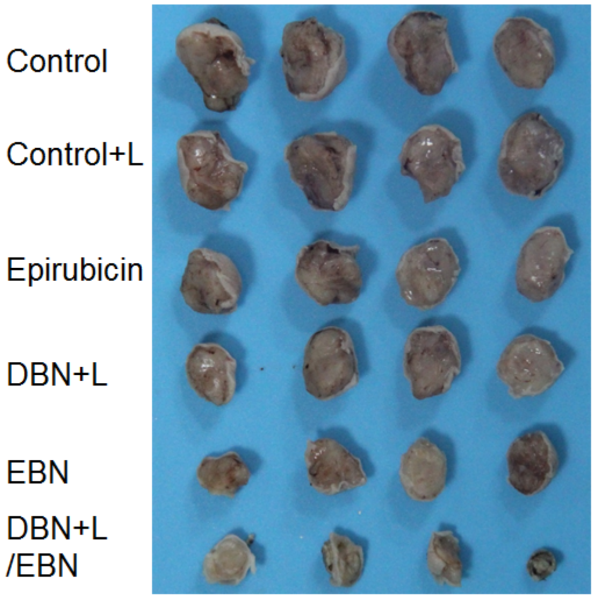

Figure S7 The typical photographs of lung tissues from each group.

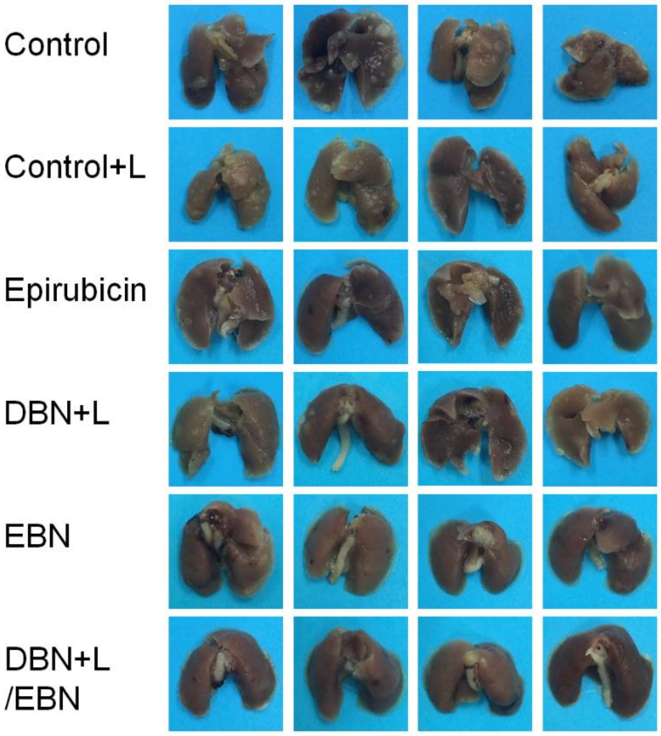

Figure S8 The typical photographs of lung tissues from each group.

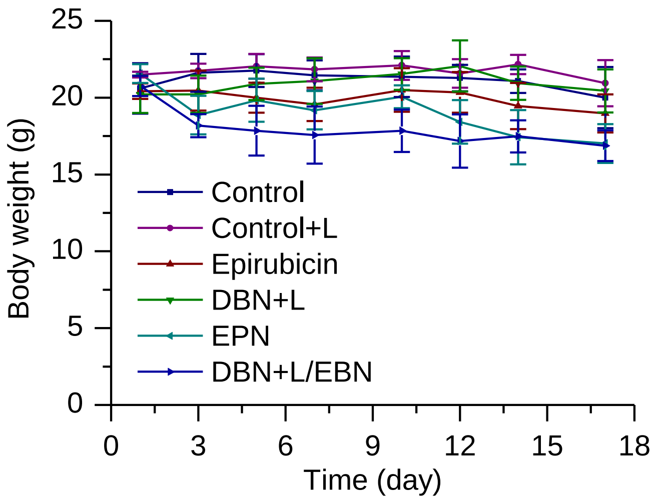

Figure S9 The body weight changes during the treatment from each group.

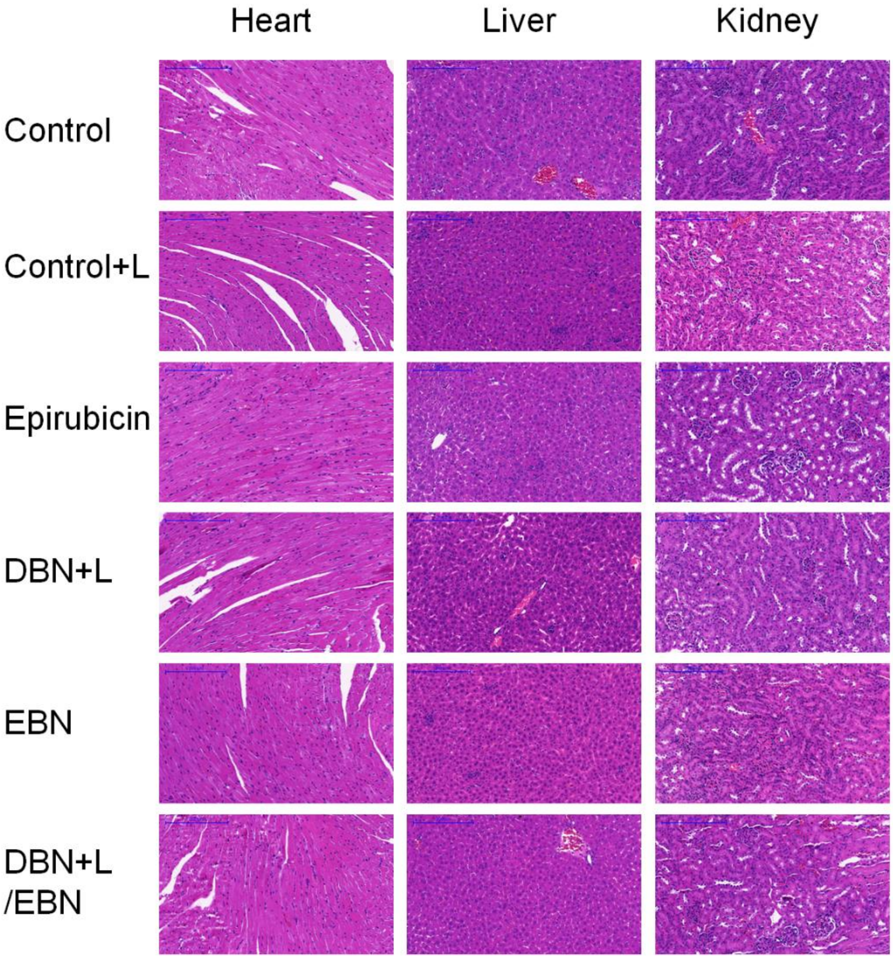

Figure S10 The histological examinations of heart, liver and kidney from each group.
